# Inwardly Rectifying Potassium Channel Kir4.1 as a Novel Modulator of BDNF Expression in Astrocytes

**DOI:** 10.3390/ijms19113313

**Published:** 2018-10-24

**Authors:** Yukihiro Ohno, Masato Kinboshi, Saki Shimizu

**Affiliations:** Department of Pharmacology, Osaka University of Pharmaceutical Sciences, 4-20-1 Nasahara, Takatsuki, Osaka 569-1094, Japan; kinboshi@kuhp.kyoto-u.ac.jp (M.K.); s.shimizu@gly.oups.ac.jp (S.S.)

**Keywords:** astrocytes, brain-derived neurotrophic factor (BDNF), depressive disorders, epilepsy, Kir4.1 channels, pain, spatial potassium buffering

## Abstract

Brain-derived neurotrophic factor (BDNF) is a key molecule essential for neural plasticity and development, and is implicated in the pathophysiology of various central nervous system (CNS) disorders. It is now documented that BDNF is synthesized not only in neurons, but also in astrocytes which actively regulate neuronal activities by forming tripartite synapses. Inwardly rectifying potassium (Kir) channel subunit Kir4.1, which is specifically expressed in astrocytes, constructs Kir4.1 and Kir4.1/5.1 channels, and mediates the spatial potassium (K^+^) buffering action of astrocytes. Recent evidence illustrates that Kir4.1 channels play important roles in bringing about the actions of antidepressant drugs and modulating BDNF expression in astrocytes. Although the precise mechanisms remain to be clarified, it seems likely that inhibition (down-regulation or blockade) of astrocytic Kir4.1 channels attenuates K^+^ buffering, increases neuronal excitability by elevating extracellular K^+^ and glutamate, and facilitates BDNF expression. Conversely, activation (up-regulation or opening) of Kir4.1 channels reduces neuronal excitability by lowering extracellular K^+^ and glutamate, and attenuates BDNF expression. Particularly, the former pathophysiological alterations seem to be important in epileptogenesis and pain sensitization, and the latter in the pathogenesis of depressive disorders. In this article, we review the functions of Kir4.1 channels, with a focus on their regulation of spatial K^+^ buffering and BDNF expression in astrocytes, and discuss the role of the astrocytic Kir4.1-BDNF system in modulating CNS disorders.

## 1. Introduction

Astrocytes are the most abundant glial cells and play important roles in maintaining the integrity of brain functions. It is known that a single astrocyte connects 10^5^ or more synapses, where they form “tripartite synapses” consisting of pre-synaptic nerve terminals, post-synaptic membranes of neurons and perisynaptic processes of astrocytes [1,2,3]. Thus, astrocytes actively regulate the excitability of neurons by regulating ion and water homeostasis at synapses, metabolizing neurotransmitters (e.g., glutamate and GABA) and secreting various neuroactive substances (e.g., gliotransmitters, neurotrophins, and cytokines) [2,3,4,5,6]. In addition, astrocytes are implicated in the pathogenesis of various disorders including schizophrenia, major depressive disorders, Parkinson’s disease, Alzheimer’s disease, epilepsy, and chronic pain [7,8,9].

Brain-derived neurotrophic factor (BDNF) is a key molecule essential for development and pathophysiology of the brain. Specifically, BDNF mediates molecular mechanisms underlying synaptic plasticity (e.g., neurite outgrowth and synaptogenesis), neural development, cell survival and active gliosis [10,11,12,13]. Elevated expression of BDNF is implicated in the pathogenesis of epilepsy and chronic pain sensitization whereas the neurotrophic property of BDNF is expected to restore neurodegenerative disorders such as Parkinson’s disease and Alzheimer’s disease. Early studies showed that BDNF was expressed in pyramidal neurons in the cerebral cortex and hippocampus [14,15], but it is now known that astrocytes are an alternative source for BDNF expression and secretion [16,17]. Furthermore, recent evidence illustrates that astrocytic BDNF expression is specifically modulated by the inwardly rectifying potassium (Kir) 4.1 channels, which mediate the spatial potassium (K^+^) buffering function of astrocytes and regulate neuronal activities [9,18].

In this article, we review the molecular and functional characteristics of Kir4.1 channels, with a focus on their actions in regulating spatial K^+^ buffering and BDNF expression in astrocytes, and discuss the role of the astrocytic Kir4.1-BDNF system in modulating central nervous system (CNS) disorders.

## 2. Astrocytic Kir4.1 Channels

### 2.1. Molecular Structure and Channel Properties

**Figure 1:**
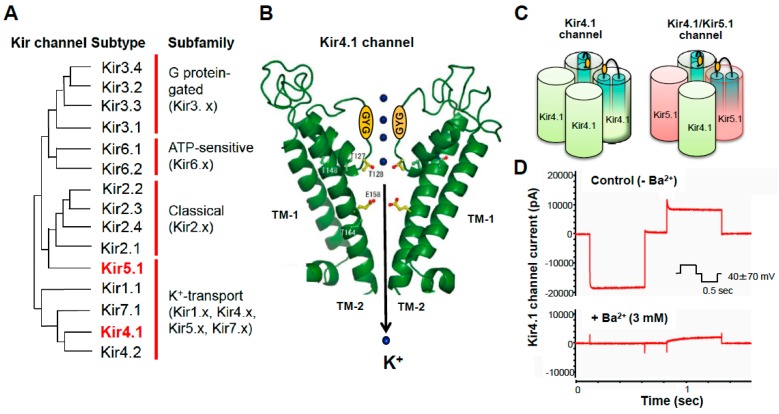
Molecular structure and channel properties of Kir4.1 channels. (**A**) Classification of Kir channels and their phylogenic tree. Kir channels consist of 15 subtypes which can be classified into seven families. (**B**) Kir4.1 subunits have two TM structures (TM-1 and TM-2) with one extracellular loop which contains a signature sequence: (GYG) participating in ion-selective filtering for K^+^. (**C**) Kir4.1 subunits form two types of Kir4.1 channels, the homo-tetramer of Kir4.1 (Kir4.1) and the hetero-tetramer of Kir4.1 and Kir5.1 (Kir4.1/5.1). (**D**) Kir4.1 channels conduct large inward and smaller outward K^+^ currents (sensitive to Ba^2+^), illustrating characteristics of inwardly rectifying K^+^ channels.

Kir channels are generally classified into seven families (Kir1 through Kir7) encompassing more than 15 members [19,20]. These can be categorized by their characteristics into four groups: (1) G protein-aged channels (Kir3.x); (2) ATP-sensitive channels (Kir6.x); (3) classical channels (Kir2.x); and (4) K^+^-transport channels (Kir1x, Kir4.x, Kir5.x, and Kir7.x) (Figure 1). Among them, Kir4.1 subunits are highly expressed in the brain astrocytes, where they mediate the spatial K^+^ buffering action of astrocytes [5,6,7,8,20,21,22,23,24,25]. Kir4.1 subunits are also expressed in retina (Müller cells) and kidney (e.g., distal tubular epithelia).

The *KCNJ10* (or *Kcnj10*) gene encoding the Kir4.1 subunit is located on chromosome (Chr) 1 (1q23.2) in humans, Chr 13 (13q24) in rats and Chr 1 (1 H33) in mice, coding 379 amino acids. Kir4.1 has two transmembrane (TM) regions with one extracellular loop which contains a signature sequence: (GYG) participating in ion-selective filtering for K^+^ (Figure 1) [5,6,8,20]. Kir4.1 channels are constructed as a homo-tetramer of Kir4.1 subunits. Kir4.1 subunits also form Kir4.1/5.1 channels (hetero-tetramer of Kir4.1 and Kir5.1 subunits) with another subunit Kir5.1, Kir4.1, and Kir4.1/5.1 channels allow large inward K^+^ currents at potentials negative to K^+^ equilibrium potential (E_K_) and moderate outward K^+^ currents at those positive to E_K_ [5,6,7,8,20]. In contrast to Kir4.1 and Kir4.1/5.1 channels, Kir5.1 channels (a homo-tetramer of Kir5.1 subunits) are normally inactive [20,26]. It is also known that polyamines (e.g., spermine) which exist in cells at relatively high concentrations, as well as Mg^2+^, are involved in the rectifying properties of Kir4.1 and Kir4.1/Kir5.1 channels by inactivating the channel gating at depolarized membrane potentials [20,21].

### 2.2. Mediation of Astrocytic K^+^ Buffering

**Figure 2:**
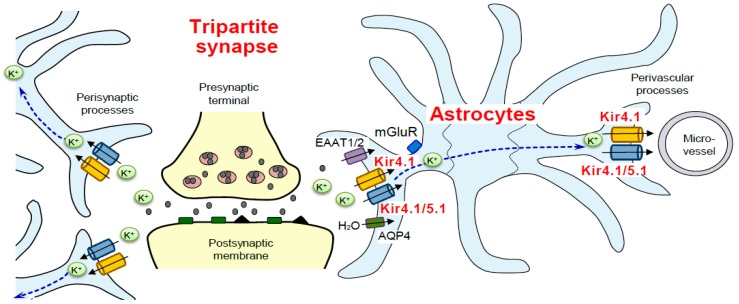
Kir4.1 channels mediate the spatial K^+^ buffering action of astrocytes. The spatial K^+^ buffering action of astrocytes is essential for controlling extracellular K^+^ concentrations at tripartite synapses. Kir4.1 and Kir4.1/5.1 channels located in perisynaptic and perivascular processes conduct the K^+^ buffering currents in astrocytes. Kir4.1 channels facilitate glutamate (Glu) and water uptake into astrocytes via coupling to excitatory amino acid transporters (EAATs) and aquaporin 4 (AQP4).

Spatial K^+^ buffering by astrocytes is essential for controlling the extracellular K^+^ concentration ([K^+^]_o_) at synapses and neuronal excitability (Figure 1) [5,6,7,8,20]. Neurons release considerable amounts of K^+^ during the action potential repolarization phase and, if uncorrected, [K^+^]_o_ elevate to 10 mM or more, which produces abnormal neuronal discharges and finally spreading depression. The spatial K^+^ buffering action of astrocytes removes excess extracellular K^+^ and transports it to regions of lower [K^+^]_o_ such as microvessels (Figure 2). The spatial K^+^ buffering system is also known to be coupled to glutamate uptake via glutamate transporters (e.g., excitatory amino acid transporter 1 (EAAT1) and EAAT2) and water transport via aquaporin-4 (AQP4) into astrocytes [5,6,7,8,20].

As described previously, spatial K^+^ buffering is primarily mediated by Kir4.1 and Kir4.1/5.1 channels which are specifically expressed in astrocytes (Figure 2). Kir4.1 and Kir4.1/5.1 channels can take up excessive extracellular K^+^ locally elevated at synapses, depending on the difference between local E_K_ and the membrane potential of astrocytes [5,6,7,8,20,21,22,23,24,25,26,27,28]. If the function of Kir4.1 or Kir4.1/5.1 channels is disrupted under certain disease conditions or by drug interactions, it increases the excitability of neurons by elevating [K^+^]_o_ and extracellular glutamate ([Glu]_o_) levels (Figure 1) [7,29].

### 2.3. Pharmacology

**Figure 3:**
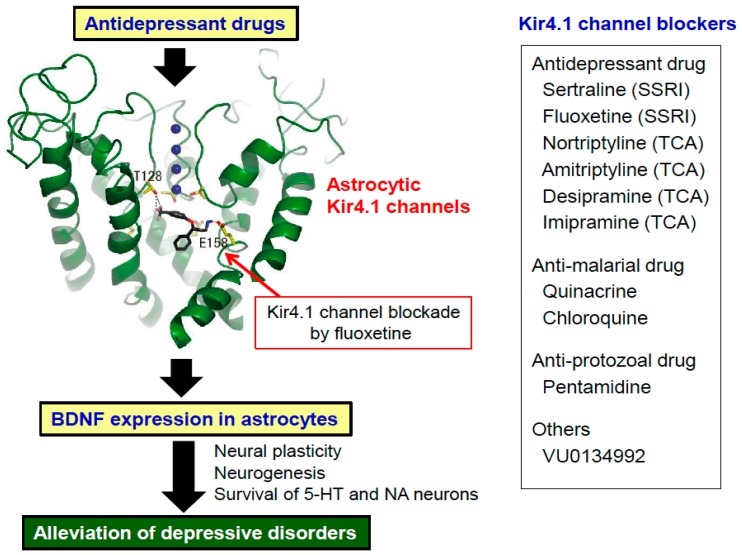
Non-monoaminergic mechanism underlying the antidepressant action of fluoxetine via astrocytic brain-derived neurotrophic factor (BDNF) expression and reported Kir4.1 channel blockers. In silico docking model analysis of Kir4.1 channels with antidepressant drugs revealed that Glu158 can interact with the amine moiety of fluoxetine with an ionic bond and Thr128 with the benzene ring (hydrogen bond acceptor) with a hydrogen bond. The blockade of Kir4.1 channels by fluoxetine facilitate a BDNF mRNA and protein expression in astrocytes, which can potentially alleviate depressive disorders by enhancing neural plasticity, neurogenesis, and survival of 5-HT and noradrenaline (NA) neurons. Kir4.1 channel blockers currently reported are shown in a right side panel. SSRI: Selective serotonin reuptake inhibitor. TCA: Tricyclic antidepressants.

Several agents are reported to act on Kir4.1 channels. After screening CNS drugs in Kir4.1-expressing HEK293 cells, we found that several antidepressant agents reversibly inhibited Kir4.1 channels in concentration- and subunit-dependent manners. Of these, tricyclic antidepressants (TCAs), amitriptyline, nortriptyline, desipramine, and imipramine, inhibited Kir4.1 channel activities in a voltage-dependent manner [30,31]. In addition, selective serotonin reuptake inhibitors (SSRIs), sertraline, fluoxetine, and fluvoxamine, also inhibited Kir4.1 channels, but in a voltage-independent manner [32]. The inhibitory action of fluoxetine was Kir4.1-specific and was not observed for Kir1.1 or Kir2.1 channels. The inhibitory concentration of fluoxetine was considered to be in a range of brain concentrations observed during the treatment of patients with depression. Furthermore, alanine-scan mutagenesis studies on the Kir4.1-drug interaction revealed that these antidepressants specifically bound to the central cavity of the channel [33]. Specifically, T128 and E158, which are located in the Kir4.1 channel pore, were identified to bind to the antidepressants. In addition, in silico docking simulation analysis illustrated that Kir4.1 channels had a pocket, where E158 interacts with the amine moiety of the antidepressant molecules with an ionic bond and T128 binds to their benzene ring (hydrogen bond acceptor) with a hydrogen bond (Figure 3). Estimated volumes of the channel’s central cavity were 220 Å^3^ and 360 Å^3^ in closed and open conformations, respectively, which can accommodate the size of the antidepressants (e.g., fluoxetine: 213 Å^3^).

Besides antidepressant drugs, several agents have been shown to interact with Kir4.1 channels. These include anti-malarial drugs, quinacrine and chloroquine [34,35], an intracellularly-acting blocker, pentamidine (anti-protozoal drug) [36], and VU0134992, a novel compound identified from the chemical library of the Vanderbilt Institute [37]. Interestingly, most Kir4.1 inhibitors (e.g., quinacrine, chloroquine, and pentamidine) were also shown to bind to T128 and E158, likely antidepressant drugs, while VU0134992 primarily interacted with 158E and 159I. Although information is still limited, these findings on the structure-activity relationship of Kir4.1 channels are important for designing new ligands for Kir4.1 or Kir4.1/5.1 channels.

### 2.4. Modulation of BDNF Expression in Astrocytes

In response to various pathophysiological stimuli, astrocytes activate diverse signal transduction pathways (e.g., Ca^2+^ signaling and multiple second messenger-protein kinase cascades) and secrete various neuroactive molecules including BDNF [38,39]. Particularly, a series of studies showed that various antidepressant agents (e.g., amitriptyline, imipramine, fluoxetine, and fluvoxamine) induced the expression of BDNF in astrocytes [40,41,42,43], suggesting that BDNF induction in astrocytes contributes to the therapeutic actions of antidepressant drugs. In addition, since both BDNF and Kir4.1 channels are expressed in astrocytes [5,6,7,8,9,16,17] and since many antidepressant drugs inhibit the Kir4.1 channels [30,31,32,33], BDNF induction by antidepressants is thought to be brought about by their blocking actions on Kir4.1 channels.

To confirm the above hypothesis, we investigated the effects of Kir4.1 channel inhibition (i.e., blockade of Kir4,1 channel activities by antidepressants and knockdown of Kir4.1 expression by siRNA transfection) on expression of neurotrophic factors in cultured astrocytes [9]. Consistent with previous studies [43], antidepressants at concentrations which inhibited Kir4.1 channel activities markedly facilitated the mRNA and protein expression of BDNF in astrocytes. In addition, relative potencies (sertraline > fluoxetine > imipramine >> fluvoxamine > mianserin) of antidepressant drugs for BDNF induction were consistent with those for the Kir4.1 channel inhibition, but not for the inhibition of serotonin reuptake (imipramine > fluoxetine = fluvoxamine > sertraline >> mianserin) [9]. This suggests that enhancement of BDNF expression was mediated by Kir4.1 channel inhibition, but not serotonin transporter inhibition. Our findings imply that the Kir4.1-BDNF interaction in astrocytes serve as a non-aminergic mechanism for the action of antidepressant drugs (Figure 3).

**Figure 4:**
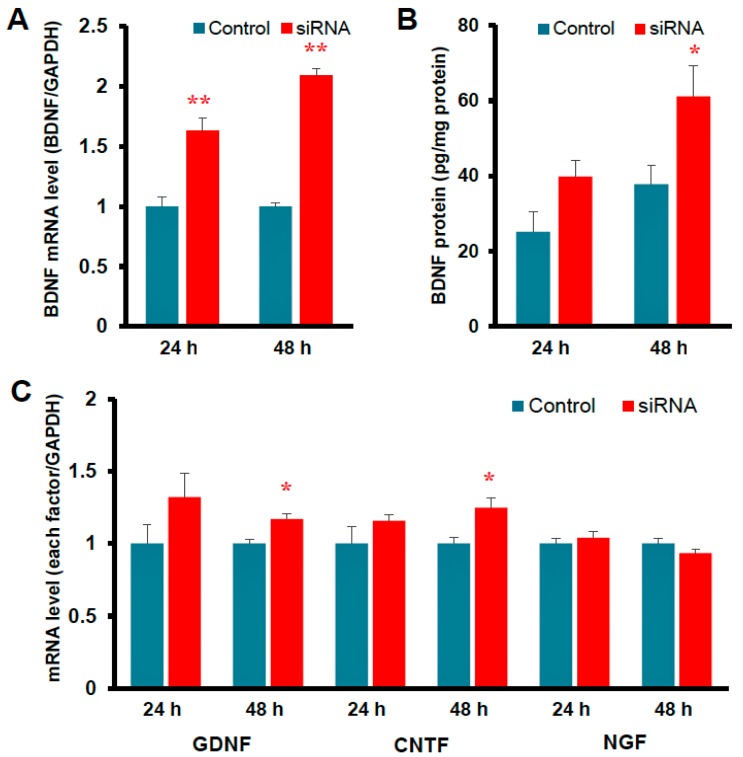
Effects of Kir4.1 knockdown (down-regulation) by the transfection of Kir4.1 siRNA on the expression of BDNF and other neurotrophic factors in astrocytes. (**A**) Effects on BDNF mRNA and (**B**) protein expression. (**C**) Effects on mRNA expression of other neurotrophic factors (GDNF, CNTF, and NGF). The mRNA and protein levels were analyzed at 24 h or 48 h after the transfection of Kir4.1 siRNA. * *p* < 0.05; ** *p* < 0.01. Graphs are quoted from *Front. Mol. Neurosci*. 2017, 10, 408 [9].

**Figure 5:**
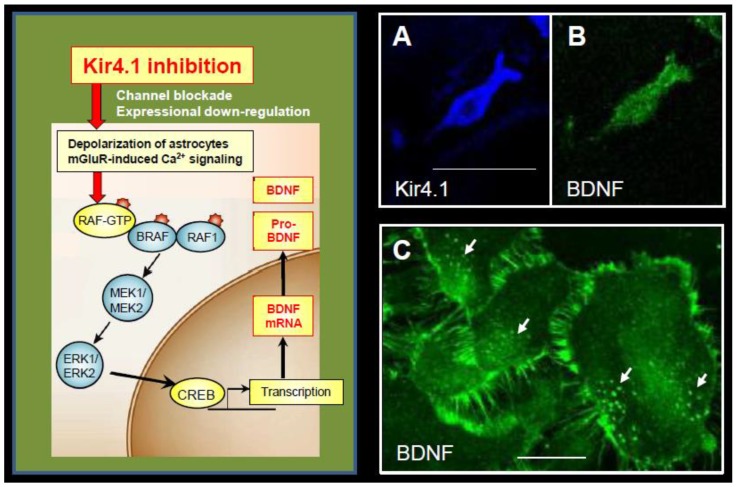
Mechanism underlying enhanced BDNF expression by Kir4.1 channels inhibition in astrocytes. Inhibition (channel blockade and expressional down-regulation) of Kir4.1 channels cause a depolarization of astrocytes (6,46) and/or activation of metabotropic glutamate receptors (mGluR) via elevating the [K^+^]_o_ and [Glu]_o_ levels at synapses, respectively [46,47]. These events subsequently activate the Ras/Raf/MEK/ERK signaling pathway and enhance BDNF expression in astrocytes. The right photographs show co-expression of Kir4.1-immunoreactivity (IR) (blue: Alexa Fluor647) (**A**) and BDNF-IR (green: Alexa Fluor488) (**B**) in the same astrocyte. (**C**) Cultured astrocytes at a confluent stage contain many BDNF-containing secretary granules (arrows), where BDNF-IR spread from the soma to fine processes surrounding astrocytes, reflecting the secretary process of BDNF. Scale bar: 25 µm (**A** and **B**) and 50 µm (**C**). Photographs are quoted from *Front. Mol. Neurosci*. 2017, 10, 408 [9].

Since the expression of Kir4.1 channels is known to be down-regulated under certain disease conditions (e.g., epilepsy), we also evaluated the effects of Kir4.1 knockdown on astrocytic BDNF expression. Inhibition of Kir4.1 expression by the transfection of Kir4.1 siRNA markedly elevated the mRNA and protein levels of BDNF (Figure 4). Knockdown of Kir4.1 expression by Kir4.1 siRNA transfection slightly, but significantly, increased the expression of glia cell line-derived neurotrophic factor (GDNF) and ciliary neurotrophic factor (CNTF), but did not affect the expression of nerve growth factor (NGF) (Figure 4). Furthermore, the BDNF induction by Kir4.1 siRNA transfection was suppressed by a MEK1/2 inhibitor, but not by a p38 MAPK or a JNK inhibitors [7]. These results strongly suggest that inhibition of Kir4.1 channels facilitates astrocytic BDNF expression by activating the Ras/ERK pathway (Figure 5). Previous studies also showed that the Ras/ERK pathway regulates the transcription of BDNF and other survival/plasticity genes through interaction with cyclic AMP response element binding protein (CREB) [44,45].

The above evidence indicates that Kir4.1 plays an important role in modulating the expression of BDNF in astrocytes. Specifically, reduced expression and inhibition of Kir4.1 channels associated with CNS diseases or drug interactions seem to facilitate the BDNF expression in astrocytes. Since inhibition of Kir4.1 channels caused a depolarization of astrocytes by accumulating intracellular K^+^ [46] and also facilitated Ca^2+^ signaling via activation of metabotropic glutamate receptors by elevated [Glu]_o_ [47], these events probably activate the Ras/ERK pathway and enhance BDNF expression (Figure 5). In addition, since antidepressants are known to increase the GDNF expression via ERK activation in glial cells [48,49,50], it is also likely that antidepressants elevate the GDNF expression by inhibiting Kir4.1 channels.

## 3. Pathogenic and Therapeutic Roles of the Kir4.1-BDNF System

### 3.1. Epilepsy

Epilepsy is a chronic neurological disease that is characterized by recurrent convulsive or non-convulsive seizures attributed to abnormal excitation of neurons [51,52]. Various antiepileptic drugs are available to treat epilepsy, which primarily act on neural components, but not glial cells. Mechanisms of current medications for epilepsy include the inhibition of voltage-gated Na^+^ channels (e.g., phenytoin, carbamazepine, and lamotrigine), the activation of GABAergic neurotransmission by inhibiting GABA transaminase (e.g., sodium valproate) or stimulating GABA_A_ receptors (e.g., phenobarbital and diazepam), the blockade of voltage-gated Ca^2+^ channels (e.g., gabapentin) or the antagonism of glutamate receptors (e.g., topiramate) [53]. These standard antiepileptic drugs can control about 70% of epilepsy patients; but not the remaining 30% of patients. These patients suffer from refractory symptoms and are often subjected to surgical treatments (e.g., ablation of seizure foci, deep brain stimulation, and vagus nerve stimulation) [54].

Evidence is accumulating that astrocytic Kir4.1 channels are closely involved in the onset and development of epilepsy (epileptogenesis). Studies using conditional knockout (cKO) animals targeted to astrocytic Kir4.1 showed a clear relationship between dysfunction of astrocytic K^+^ buffering and seizure sensitivity (Table 1) [29]. Kir4.1-cKO mice developed marked body tremor and ataxia, and showed hypersusceptibility to seizure generation. In humans, loss-of-function mutations in the *KCNJ10* gene encoding Kir4.1 cause epileptic disorders, “EAST” or “SeSAME” syndrome [55,56]. Patients with “EAST” and “SeSAME” syndromes show mostly identical symptoms including generalized tonic-clonic seizures, ataxia, sensorineural deafness and renal tubulopathy (Table 2). Frequent mutations of *KCNJ10* in the EAST/SeSAME syndrome were R65P at the cytoplasmic end of the TM-1 region, G77R at TM-1, C140R at an extracellular loop between TM-1 and TM-2, T164I, and A167V at the cytoplasmic end of TM-2, R175Q, R199X, and R297C at the C-terminal domain [26,57,58]. All these mutations caused a drastic reduction in K^+^ buffering currents mediated not only by Kir4.1 channels, but also by Kir4.1/5.1 channels, illustrating that Kir4.1 is essential for the function of both Kir4.1 and Kir4.1/5.1 channels. Knockdown of Kir4.1 expression by cKO or siRNA transfection reportedly impaired K^+^- and glutamate-uptake into astrocytes, elevating [K^+^]_o_ and [Glu]_o_ [29,59]. Seizure-susceptible DBA/2 mice carry the T262A mutation in the *Kcnj10* gene and show impaired Kir4.1 channel activity and reduced glutamate clearance by astrocytes in the hippocampus (Table 1) [60,61]. Therefore, it is likely that dysfunction (e.g., gene mutation, reduced expression, and pharmacological blockade) of Kir4.1 channels disrupts the spatial K^+^ buffering action of astrocytes and increases the [K^+^]_o_ and [Glu]_o_ levels, which evokes epileptic seizures (ictogenesis) (Figure 3). The deafness and abnormal renal excretion of electrolytes in the EAST/SeSAME syndrome (Table 2) seem to be attributable to Kir4.1 dysfunction in the inner ear and renal epithelial cells, respectively, since Kir4.1 channels are involved in the maintenance of endocochlear potential and the electrolytes transport (e.g., K^+^) in the scala media and the distal convoluted tubules [55,56].

Regarding the pathophysiological alterations of Kir4.1 channels in epilepsy, previous studies showed that brain Kir4.1 expression was down-regulated in animal models of convulsive seizures, including DBA/2 mice [60,61], Noda epileptic rats (NER) [62], a post-traumatic epilepsy model [63], and an albumin-induced seizure model in rats [64], but not in a model of absence seizures (Groggy rats) (Table 1) [65]. NER showed a region-specific reduction in the Kir4.1 channel expression in the amygdala region (Table 1) [62], which is closely related to epileptogenesis and human temporal lobe epilepsy (TLE) [66,67]. Interestingly, the loss of Kir4.1 channels was specifically observed in the perisynaptic processes of astrocytes surrounding the amygdala neurons whereas Kir4.1 levels in the astrocyte somata were not altered. These findings imply that the reduced expression (down-regulation) of Kir4.1 channels impaired the K^+^ buffering action of astrocytes and caused hyperexcitation of amygdala neurons in NER, which led to induction of generalized tonic-clonic seizures. In humans, several clinical studies showed the down-regulation and impaired functioning of Kir4.1 channels in patients with TLE [8,68,69,70,71], suggesting a causative role of Kir4.1 channels in TLE. In addition, single nucleotide polymorphisms of the *KCNJ10* gene have been shown to be associated with epileptic disorders (e.g., TLE accompanying febrile seizures and childhood epilepsy) [72,73,74].

Besides changes in Kir4.1 channels, BDNF expression is also known to be altered in epileptic disorders. Numerous studies showed that BDNF expression was up-regulated in various animal models of epilepsy [10,11,12,13,75,76,77] and in human epileptic disorders [78,79]. Although most of these studies did not differentiate neurons or astrocytes as source cells expressing BDNF, a recent study showed that up-regulation of BDNF expression occurred both in neurons and astrocytes during epileptogenesis [80]. These findings support the concept that inhibition of Kir4.1 channels, which occurs under certain epileptic conditions, facilitates the BDNF expression in astrocytes.

**Figure 6:**
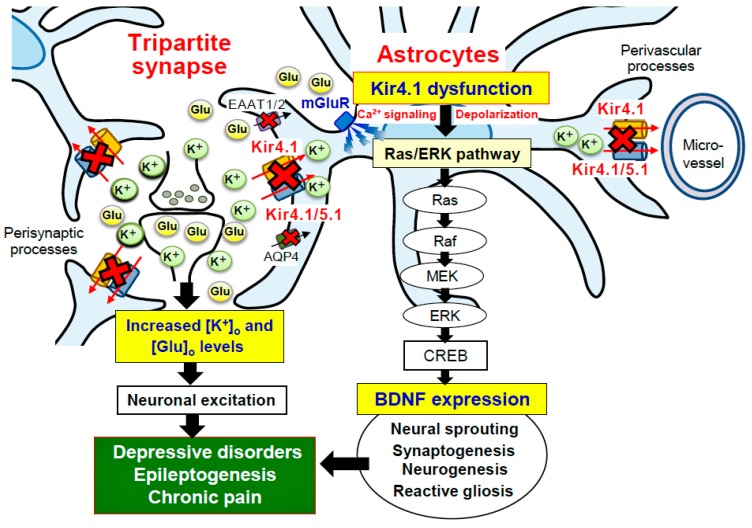
Schematic diagram showing the effects of Kir4.1 inhibition on neuronal excitability and astrocytic BDNF expression. Inhibition (e.g., gene mutation, reduced expression, and pharmacological blockade) of Kir4.1 channels increases the [K^+^]_o_ and [Glu]_o_ levels at synapses and elevates neural excitability. Kir4.1 inhibition also activates the Ras/Raf/MEK/ERK signaling pathway and enhances BDNF expression in astrocytes, facilitating neural sprouting, synaptogenesis, neurogenesis and reactive gliosis. Through these influences, Kir4.1-containnng channels seem to play crucial roles in modulating the development of central nervous system disorders such as epilepsy and mood disorders (e.g., major depression).

BDNF is known as a key mediator of epileptogenesis, eliciting synaptic plasticity, neural sprouting, neurogenesis, and reactive astrogliosis [76]. Knockdown of the BDNF gene has been shown to prevent the development of epilepsy [10,11,12,13]. In addition, inhibition of the BDNF receptor TrkB has been shown to suppress seizure development in TLE models and ameliorate status epilepticus-induced chronic recurrent seizures [75,77]. It is therefore likely that the astrocytic Kir4.1-BDNF system plays a crucial role in facilitating epileptogenesis (development of chronic epilepsy) (Figure 6). Conversely, enhancement of Kir4.1 channel expression or the Kir4.1 channel activity may cure or prevent epileptic disorders. Supporting the latter hypothesis, we recently showed that repeated treatments with antiepileptic drugs effective for generalized tonic-clonic seizures (valproate, phenytoin, and phenobarbital) commonly elevated the astrocytic Kir4.1 channel expression in the limbic regions. These actions may be at least partly related to their antiepileptic actions [81].

### 3.2. Depressive Disorders (DDs)

Nearly 20% of the population experiences a major depressive episode which is characterized by a blunted mood and an inability to experience pleasure (anhedonia) [82]. DDs (e.g., major depression, depressive mood and dysthymia) are complex psychiatric disorders with diverse symptoms that not only negative affect mood and behavior, but also cognitive impairments, sleep disturbances and somatic symptoms. It is well documented that development of DDs is associated with a reduction in monoamines (especially 5-HT and noradrenaline) in the brain [83]. In addition, numerous studies have shown that patients with DDs have accompanying morphological deficits (e.g., dendritic retraction and atrophy) in the brain regions (e.g., hippocampus, amygdala, and prefrontal cortex) regulating mood, behavior, and cognition. To treat DDs, antidepressants which enhance the activities of noradrenaline and 5-HT neurons are widely used, including TCAs (e.g., nortriptyline, clomipramine, and imipramine), SSRIs (e.g., fluoxetine, sertraline, and paroxetine) and serotonin noradrenaline reuptake inhibitors (SNRIs) (e.g., milnacipran, duloxetine and venlafaxine), noradrenergic and specific serotoninergic antidepressants (NaSSAs) (e.g., mirtazapine) [84]. Most of these antidepressants, except for NaSSAs, commonly bind to noradrenaline and/or 5-HT transporters and inhibit their reuptake into the axon terminals, elevating the noradrenaline and/or 5-HT levels at the synaptic cleft. SSRIs, SNRIs, and NaSSA have a superior safety profile to that of the classical antidepressants (e.g., TCAs) that cause anti-cholinergic (e.g., constipation and dry mouth), anti-histamine (e.g., sedation), anti-adrenergic (e.g., orthostatic hypotension, tachycardia, and sedation) and cardiotoxic (e.g., fatal arrhythmia) side effects. Nonetheless, there still exist clinical unmet needs in the treatment of DDs including: (1) a reduction in gastrointestinal side effects (e.g., nausea and vomiting); (2) rapid onset of antidepressant action; and (3) sufficient efficacy for severe depression and/or refractory symptoms.

There is no clinical study so far on the pathophysiological alterations of Kir4.1 channels in human DDs. Nonetheless, previous studies on the actions of antidepressant drugs in astrocytes [40,85], especially on their interactions with Kir4.1 channels [9,18,30], yield a hypothesis that Kir4.1 channels are implicated in modulating DDs [18,31]. Potential mechanisms of Kir4.1 channels underlying the modulation of DDs can be explained from two points of view: (1) regulation of neuronal excitability via spatial K^+^ buffering and (2) regulation of BDNF expression in astrocytes. Inhibition (down-regulation or blockade) of Kir4.1 channels attenuates K^+^ buffering, increases neuronal excitability by elevating [K^+^]_o_ and [Glu]_o_ levels and facilitates BDNF expression, which seem to improve DDs (Figure 3 and Figure 6). Conversely, activation (up-regulation or opening) of Kir4.1 channels reduces neuronal excitability by lowering [K^+^]_o_ and [Glu]_o_ levels and attenuates BDNF expression, which may cause DDs. Interestingly, a recent study by Cui et al. (2018) demonstrated that astrocytic Kir4.1 channels in the lateral habenula closely regulate the expression of neuronal bursting and depressive behaviors in rats [86]. They showed that overexpression (gain-of-function) of astrocytic Kir4.1 channels increased neuronal bursts in the habenula and produced depression-like behaviors (i.e., immobility in a forced swim test and reduced sucrose preference), whereas expressional knockdown (loss-of-function) of Kir4.1 channels in the habenula decreased bursting and rescued depression-like behaviors.

BDNF has long been implicated in the development of DDs. It is known that DDs are associated with morphological degradation (e.g., dendritic retraction, decreased neurogenesis, and atrophy) of the limbic structures [87,88,89]. These events are thought to be caused by metabolic disturbances or reduced expression of BDNF especially under exposure to chronic stress [87,89]. Multiple missense polymorphisms of the *BDNF* gene are also reported to be associated with DDs [89,90]. Moreover, antidepressant drugs increase both neural and astrocytic BDNF expression in the hippocampus and prefrontal cortex [41,42,43,87,91,92,93,94,95,96] and rescue impaired neurogenesis in the hippocampus [93,97,98]. Deletion of TrkB blocks antidepressant-induced neurogenesis [99], supporting a role for BDNF in these actions. Histopathological studies also showed that the antidepressant treatment elevates the level of BDNF and facilitates neurogenesis in the hippocampus of the patients with DDs [100,101]. Similarly, serum BDNF levels [102] and hippocampal volume [103,104] are partially restored by the antidepressant treatment in depressed patients. All these findings reveal that BDNF is a key molecule in modulating the pathogenesis and treatment of DDs. In addition, since many antidepressant drugs facilitate BDNF expression in astrocytes by inhibiting Kir4.1 channels, astrocytic Kir4.1 channels seem to be a novel therapeutic target for DDs (Figure 3 and Figure 6).

### 3.3. Other CNS Disorders

Besides epilepsy and DDs, BDNF is also implicated in the pathogenesis of pain and neurodegenerative disorders. Several studies have shown that Kir4.1 channels expressing the satellite glial cells surrounding sensory neurons were reduced in a chronic pain model [105] and in a herpetic neuralgia model [106]. In addition, knockdown of Kir4.1 using RNA interfering techniques in the trigeminal ganglion evoked facial pain-like behaviors [106]. Since BDNF is known to elicit hyperalgesia [107,108] and to be upregulated in a neuropathic pain model [109], the glial Kir4.1-BDNF system also seems to be involved in chronic pain sensitization in neuropathic pain.

Regarding neurodegenerative disorders, astrocytic Kir4.1 channels are implicated in the onset and progression of Huntington’s disease [47]. A previous study showed that expression of astrocytic Kir4.1 channels was down-regulated in Huntington’s disease models, leading to in vivo elevated extracellular K^+^ levels in the striatum [110]. In addition, viral delivery of Kir4.1 channels to striatal astrocytes alleviated motor deficits, indicating deficits of astrocytic Kir4.1 channels contribute to neural dysfunction in Huntington’s disease. However, BDNF transcription mediated by huntingtin, a protein mutated in Huntington’s disease, was reportedly reduced in Huntington’s disease models [111]. Thus, the relationship between Kir4.1 channel deficits and BDNF expression in astrocytes remains controversial in Huntington’s disease. Moreover, since pathophysiological changes in astrocytic Kir4.1 channels were also demonstrated in Alzheimer’s disease [112], Parkinson’s disease [113,114] and amyotrophic lateral sclerosis [115,116], further studies are required to elucidate the role of the astrocytic Kir4.1-BDNF system in modulating these diseases.

## 4. Closing Remarks

Expression of BDNF in astrocytes is now receiving a lot of attention as an important modulator of CNS disorders. Particularly, astrocytic Kir4.1 channels seem to play a crucial role in modulating BDNF expression. Evidence is accumulating that inhibition of Kir4.1 channels attenuates K^+^ buffering, increases neuronal excitability and enhances BDNF expression. These events seem to facilitate neural plasticity and cause sensitization, which is implicated in epilepsy and neuropathic pain. In contrast, activation of Kir4.1 channels reduces neuronal excitability by lowering [K^+^]_o_ and [Glu]_o_ levels and attenuates BDNF expression. This seems to be involved in the stress-induced atrophy of the limbic structures and the development of DDs. Since BDNF plays a key role in neural plasticity, development, and cell survival by facilitating synaptogenesis, neural sprouting, neurogenesis, and reactive gliosis, the astrocytic Kir4.1-BDNF system is expected to serve as a novel target for the treatment of CNS disorders.

## Figures and Tables

**Table 1:** Pathophysiological alterations of astrocytic Kir4.1 channels in animal models of epilepsy.

Animal Models	Changes in Kir4.1 Function and Expression	Behavioral Symptoms
cKO mice of astroglial Kir4.1	Deletion of Kir4.1 in astrocytes Inhibited Kir4.1 channel activity Reduced K^+^ and glutamate uptake by astrocytes	Tremor, ataxia, increased sensitivity to stimuli-induced GTCS, premature death
Seizure-susceptible DBA/2 mice	SNP with T262S variation in *Kcnj10* Reduced Kir4.1 channel activity and glutamate uptake by astrocytes	Increased seizure sensitivity
Noda epileptic rats (NER)	Down-regulation of Kir4.1 expression in astroglial processes in the amygdala	Spontaneous GTCS
Post-traumatic epilepsy model	Down-regulation of Kir4.1 expression in astroglial processes in the neocortex	Spontaneous partial seizures of neocortex origin
Albumin-induced seizure model in rats	Down-regulation of Kir4.1 expression in the hippocampus	Hippocampal seizures
Groggy rats (Absence seizure model)	No change in Kir4.1 expression	Absence-like seizures and ataxia

cKO, Conditional knockout; GTCS, Generalized tonic-clonic seizures; SNP, Single nucleotide polymorphism.

**Table 2:** Clinical features of EAST/SeSAME syndrome associated with *KCNJ10* mutations in humans.

	EAST Syndrome	SeSAME Syndrome
*KCNJ10* mutation	R65P, G77R, R175Q, G65P/R199X	R65P/R199X, A167V/R297C, C140R, T164I or deletion
Kir4.1 channel function	Loss of function (Partial/Total)	Loss of function
Seizure type	Generalized tonic-clonic seizures	Generalized tonic-clonic seizures
Seizure onset	3–5 months old	3–4 months old
Antiepileptic drugs used	Sodium valproate Phenobarbital Lamotrigine	Phenobarbital Phenytoin
Other symptoms	Ataxia Sensorineural deafness Tubulopathy and electrolyte imbalance (hypokalemia, alkalosis, increased K^+^, Na^+^ and Mg^2+^ excretion)	Sensorineural deafness Ataxia Mental retardation Tubulopathy and electrolyte imbalance (hypokalemia, alkalosis, increased K+, Na+ and Mg^2+^ excretion)
First report	*N. Engl. J. Med*. 2009, 360, 1960–1970 [55]	*Proc. Natl. Acad. Sci. USA* 2009, 106, 5842–5847 [56]

## Abbreviations

AQP4	Aquaporin-4
BDNF	Brain-derived neurotrophic factor
Chr	Chromosome
CNS	Central nervous system
CNTF	Ciliary neurotrophic factor
DDs	Depressive disorders
EAAT	Excitatory amino acid transporter
EAST	Epilepsy, ataxia, sensorineural deafness and tubulopathy
E_k_	K^+^ equilibrium potential
GDNF	Glia cell line-derived neurotrophic factor
[Glu]_o_	Extracellular glutamate concentration
Kir	Inwardly rectifying potassium
[K^+^]_o_	Extracellular K^+^ concentration
NaSSAs	Noradrenalinergic and specific serotonergic antidepressants
NER	Noda epileptic rats
NGF	Nerve growth factor
SeSAME	Seizures, sensorineural deafness, ataxia, mental retardation and electrolyte imbalance
SNRIs	Serotonin noradrenaline reuptake inhibitors
SSRIs	Selective serotonin reuptake inhibitors
TCAs	Tricyclic antidepressants
TLE	Temporal lobe epilepsy
TM	Transmembrane
